# Day 8 monocyte and lymphocyte counts as a novel and early predictor for discontinuation in gynecologic cancer patients receiving paclitaxel and carboplatin therapy: a retrospective observational study

**DOI:** 10.1186/s40780-026-00547-5

**Published:** 2026-02-04

**Authors:** Hiroaki Watanabe, Keita Hirai, Hirofumi Komachiya, Takafumi Naito

**Affiliations:** 1 Department of Pharmacy, Shinshu University Hospital, 3-1-1 Asahi, Matsumoto, Nagano, 390-8621 Japan; 2 Department of Clinical Pharmacy and Healthcare Sciences, Faculty of Pharmacy, Institute of Medical, Pharmaceutical and Health Sciences, Kanazawa University, 13-1 Takaramachi, Kanazawa, Ishikawa 920-8641 Japan; 3 Department of Clinical Pharmacology and Therapeutics, Shinshu University Graduate School of Medicine, 3-1-1 Asahi, Matsumoto, Nagano, 390-8621 Japan; 4 Department of Pharmacy, Showa Inan General Hospital, Akaho 3230, Komagane, Nagano, 399-4117 Japan

**Keywords:** Gynecological cancer, Paclitaxel, Carboplatin, Risk factor, Myelosuppression

## Abstract

**Background:**

Maintaining optimal treatment intensity is essential for therapeutic efficacy in gynecological malignancies undergoing paclitaxel plus carboplatin (TC) chemotherapy; however, myelosuppression frequently results in treatment delay or interruption, which can compromise long-term outcomes. While previous research has predominantly focused on pre-treatment laboratory values and late-onset adverse events, there is a crucial gap in identifying early, actionable biomarkers. This study sought to evaluate the utility of post-treatment values measured at an early time point (Day 8) to facilitate timely and proactive supportive interventions.

**Methods:**

This retrospective analysis included 242 patients who underwent TC chemotherapy. Patients who discontinued therapy for reasons other than myelosuppression (e.g., allergic reactions or patient choice) were excluded from the final analyses. The primary outcome was treatment interruption (delay or cessation) due to persistence of myelosuppression during the first cycle. Predictive modeling assessed baseline, day 8, and combined hematological factors (including neutrophil, monocyte, and lymphocyte counts, and their derived ratios). Predictive performance was evaluated using multivariate logistic regression and receiver operating characteristic (ROC) curve analyses.

**Results:**

The model incorporating both pre- and post-treatment laboratory values demonstrated superior predictive performance, evidenced by a significantly higher area under the ROC curve than the model utilizing only pre-treatment factors (0.82 vs. 0.73, *P* = 0.011). Decision tree analysis identified critical cutoff points for post-treatment laboratory values: monocyte counts below 51/µL and lymphocyte counts below 994/µL on day 8 after chemotherapy. Additionally, a pre-treatment neutrophil count of 2,795/µL was identified as a significant risk factor.

**Conclusions:**

Early post-treatment hematological indices, particularly the day 8 monocyte and lymphocyte counts, were identified as more robust predictors of chemotherapy interruption due to myelosuppression, in addition to pre-treatment indices. These findings suggest that routine monitoring and proactive supportive interventions guided by these specific early post-treatment markers should be implemented in clinical pharmacy practice to enhance treatment continuity and optimize patient outcomes.

## Background

Gynecologic malignancies, which originate from the female reproductive system, are predominantly classified as cervical, endometrial (uterine body), and ovarian cancers. Surgical resection is the primary approach for the therapeutic management of these cancers. In cases where chemotherapy is necessitated during the perioperative period or in instances of advanced recurrence, the standard treatment protocol involves TC therapy, a combination regimen of paclitaxel and carboplatin.

Although postoperative chemotherapy has proven effective [1], it is often associated with adverse effects such as myelosuppression. This complication can lead to treatment interruptions or delays, ultimately resulting in a reduction in relative dose intensity (RDI). Importantly, maintaining a high RDI is correlated with improved disease-free and overall survival in various cancers [2–4], highlighting the importance of minimizing treatment interruptions and dose reductions during chemotherapy. In this context, primary prophylactic administration of polyethylene glycol-conjugated granulocyte colony-stimulating factor (Peg-G-CSF) has been demonstrated to significantly reduce the incidence of febrile neutropenia (FN) and grade 4 neutropenia in patients receiving doxorubicin and cisplatin for uterine cancer without compromising RDI [5]. Therefore, prompt and effective supportive care is crucial for sustaining the treatment intensity.

Current knowledge regarding risk factors predominantly centers on baseline laboratory values collected before treatment [6]. However, the potential of post-treatment laboratory data to serve as predictive markers for early intervention has not yet been comprehensively investigated in gynecologic malignancies. Crucially, identifying a reliable predictor around Day 8 is clinically significant, as this time point allows for effective risk stratification before the planned Day 21 cycle, enabling proactive interventions such as timely G-CSF administration. Establishing whether post-treatment laboratory parameters, specifically those available on Day 8, function as superior risk factors could facilitate the determination of the ideal intervention timing and support the maintenance of treatment intensity. This retrospective study aimed to investigate the utility of laboratory values obtained early in treatment (Day 8), in conjunction with pre-treatment values and patient characteristics, to identify the risk factors contributing to the postponement of TC therapy due to myelosuppression. The outcomes of this study are expected to guide the development of effective monitoring strategies and be implemented in clinical pharmacy practice, thereby minimizing the occurrence of treatment interruptions and dose reductions in patients undergoing TC therapy for gynecological cancers.

## Methods

### Study design and patients

**Fig. 1:**
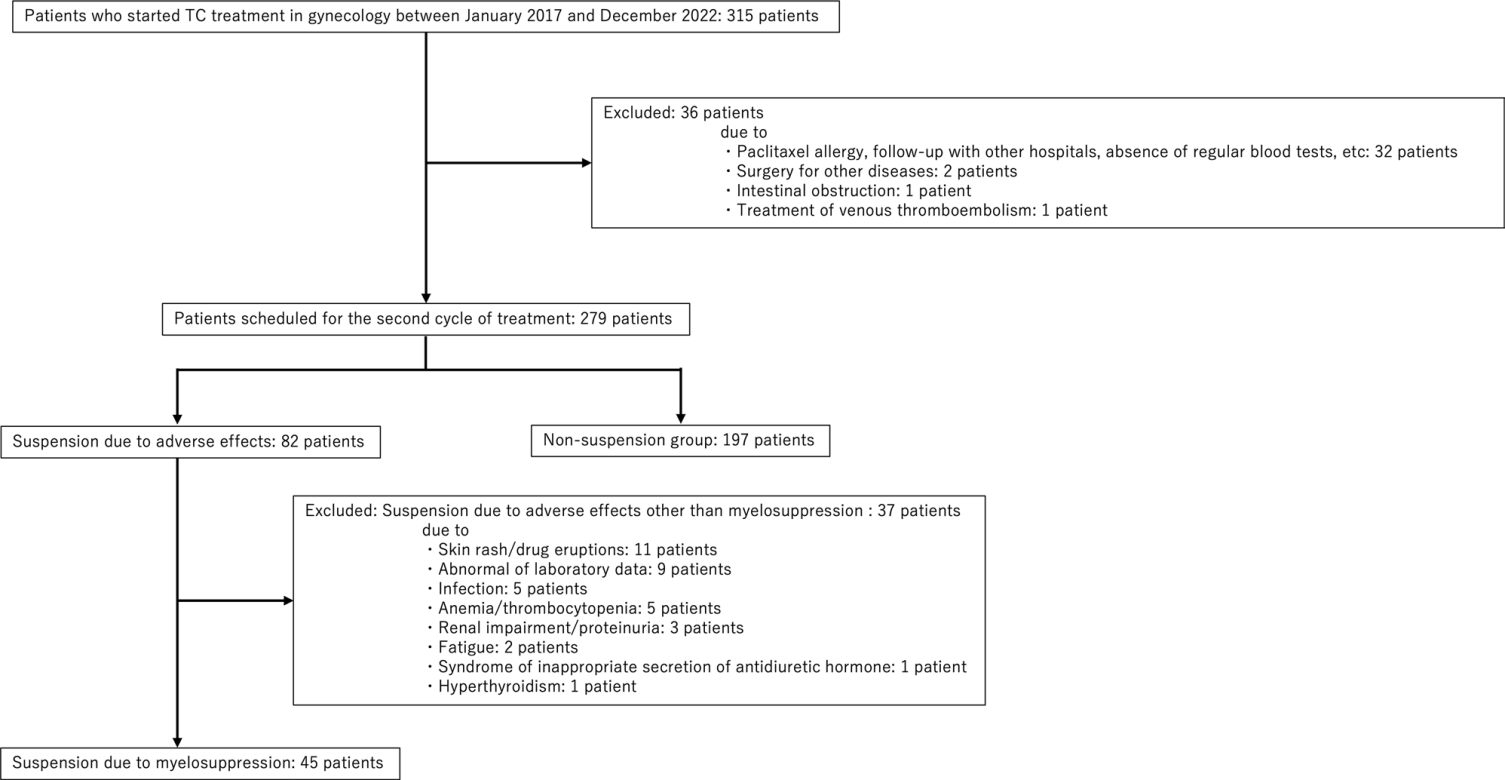
Flow diagram of patient enrollment

This retrospective study included 315 patients aged ≥ 18 years who underwent TC therapy at the Department of Obstetrics and Gynecology of Shinshu University Hospital between January 2017 and December 2022. The TC therapy regimen consisted of paclitaxel (175 mg/m²) and carboplatin (AUC = 6) administered on day 1 of each 3-week cycle. Patients were excluded from the analysis if they were unable to receive the full dose of anticancer drugs in the initial cycle owing to an allergic reaction or other medical reasons, if they had not undergone scheduled blood tests, if they had missing pre-treatment laboratory values included in the analysis, if they discontinued treatment for reasons unrelated to adverse events (e.g., when treatment for other diseases was prioritized), or if they discontinued treatment due to side effects other than myelosuppression (e.g., anemia, liver dysfunction, and other abnormal laboratory values). The flow diagram illustrates the selection of patients for analysis (Fig. 1). None of the patients received prophylactic G-CSF, nor did any receive G-CSF before day 15. This study was approved by the Ethics Committee of Shinshu University Hospital (approval number: 5833).

### Clinical data and outcomes

Necessary data were extracted from the patients’ medical records. For day 8 laboratory values, a tolerance period of ± 3 days was established to accommodate variations in outpatient examination schedules at our hospital’s Department of Obstetrics and Gynecology. This window was selected to reflect real-world clinical practice where strict scheduling is not always feasible. Patients with missing data or measurements falling outside this specified period were excluded from the analysis. The primary objective of this study was to identify the risk factors associated with patients who experienced a delay exceeding one week in commencing the second cycle of chemotherapy due to myelosuppression during the first cycle. The rationale for selecting a one-week or longer delay as the primary endpoint is based on both institutional practice and clinical prognosis. In our hospital’s Department of Obstetrics and Gynecology, TC therapy is primarily administered on fixed days of the week. Consequently, unless special circumstances arise, treatment suspensions typically result in a rescheduling to the following week (a 7-day delay). Clinically, a 2-week suspension reduces the RDI to approximately 67%. As indicated in previous studies [2–4], an RDI below 70% in ovarian cancer patients is associated with a poorer prognosis. Therefore, preventing delays from extending to two weeks is crucial for maintaining therapeutic efficacy. We selected a one-week delay (the minimum unit of suspension in our practice) as the primary endpoint to identify factors that lead to any initial disruption in the treatment schedule, thereby helping maintain an optimal RDI. The criteria for the administration of TC therapy at our hospital required a neutrophil count of at least 1,500/µL and a platelet count of at least 75,000/µL on the day of treatment. The final decision to suspend treatment based on these criteria was made at the discretion of each physician. Adverse effects were evaluated using the Common Terminology Criteria for Adverse Events (CTCAE) version 5.0.

### Statistical analysis

Fisher’s exact test was used to evaluate the statistical significance of the categorical variables, whereas the Wilcoxon rank-sum test was used for the continuous variables. Continuous variables are expressed as medians and interquartile ranges, and categorical variables are expressed as numbers and percentages. Logistic regression analysis was performed to identify factors associated with treatment suspension due to myelosuppression. In this analysis, variables with a *P* value < 0.1 in the comparison between the treatment suspension and non-suspension groups were considered candidate factors, and covariates were selected using a stepwise method. Recursive partitioning and regression tree analysis was performed based on the Classification and Regression Tree (CART) algorithm using the ‘rpart’ package in R. The model incorporated laboratory values from both pre-treatment and Day 8 as explanatory variables. The Gini index was utilized as the splitting rule to maximize node purity. To prevent overfitting and optimize the model, the following parameters were applied: the minimum number of observations required to attempt a split was set to 20, and the minimum number of observations in a terminal node was set to 7. The model complexity was optimized using 10-fold cross-validation, and the final tree was selected by pruning based on the complexity parameter (cp.) that minimized the cross-validation error. DeLong analysis was used to compare the area under the curve (AUC) values obtained from receiver operating characteristic (ROC) analysis. All statistical analyses were conducted using R software (version 4.5.0, R Foundation for Statistical Computing). Statistical significance was set at *P* < 0.05.

## Results

### Patient characteristics

Among the 315 patients who received TC therapy during the study period, excluding those who met the exclusion criteria, 197 proceeded to the second cycle of treatment without delay. In contrast, 45 patients experienced suspension of their second treatment cycle due to myelosuppression (Fig. 1). 122 patients experienced grade 3 or 4 neutropenia and 3 patients developed FN during the first cycle. Patient characteristics at the initiation of TC therapy were compared between the groups with and without treatment suspension (Table 1). The treatment suspension group tended to have a higher median age, although this was not statistically significant. No other significant differences in patient characteristics were observed between the two groups.

**Table 1:** Patient characteristics

	Suspension (*n* = 45)	Non-suspension (*n* = 197)		*P*
Age (years)	58 (54, 65)	55 (50, 65)		0.096
BMI (kg/m^2^)	22.6 (20.6, 26.5)	23.1 (20.2, 26.5)		0.258
Histology				0.911
Ovarian cancer	17 (37.8)	83 (42.1)		
Endometrial cancer	21 (44.2)	87 (44.2)		
Cervical cancer	6 (10.7)	21 (10.7)		
Other cancer	1 (2.2)	6 (3.0)		
Purpose of chemotherapy				0.653
Perioperative chemotherapy	37 (82.2)	167 (84.8)		
Recurrence or unresectable cancer	8 (17.8)	30 (15.2)		
Stage				0.378
I	17 (37.8)	64 (32.5)		
II	3 (6.7)	24 (12.2)		
III	12 (26.7)	66 (33.5)		
IV	7 (15.6)	15 (7.6)		
Recurrence	6 (13.3)	28 (14.2)		
Dose of chemotherapy (%)				
Paclitaxel	100 (100, 100)	100 (100, 100)		0.278
Carboplatin	100 (100, 100)	100 (100, 100)		0.251
Previous chemotherapy or radiation therapy history	14 (31.1)	41 (20.8)		0.138

Data are presented as medians (interquartile range) or frequencies (percentage)

BMI, body mass index

### Association between treatment suspension and laboratory values pre- and post-treatment on day 8

We performed a comparative analysis of laboratory values before treatment and on day 8 post-treatment between patients who experienced treatment suspension and those who did not (Table 2). In the pre-treatment phase, patients with treatment suspension exhibited significantly lower neutrophil, lymphocyte, and monocyte counts, along with markedly reduced hemoglobin levels. Additionally, a downward trend in platelet count was observed in the treatment suspension group. On day 8 post-treatment, laboratory values demonstrated a statistically significant decrease in neutrophil, lymphocyte, monocyte, and platelet counts in the treatment suspension group. However, there was no significant difference between the two groups in the change in values from pre-treatment to day 8 post-treatment. In this study, no patients received prophylactic G-CSF before day 15. The analysis revealed that patients who required therapeutic G-CSF after day 15 had a significantly higher rate of treatment suspension compared to those who did not. This finding suggests that relying on reactive intervention at day 15 is often insufficient to prevent treatment delays, as bone marrow suppression is likely too severe to recover in time for the next cycle. Consequently, we focused on day 8 laboratory values to identify a window for earlier, proactive intervention.

**Table 2:** Comparison of laboratory data between patients with and without treatment suspension

	Suspension (*n* = 45)		Non-suspension (*n* = 197)				*P*
**Pre-treatment laboratory values**							
Albumin (g/dL)	4.1 (4.0, 4.3)		4.2 (3.9, 4.4)			0.747	
Ccr (mL/min)	79.7 (66.6, 94.3)		85.1 (71.4, 104.7)			0.165	
AST (IU/L)	18 (15, 22)		17 (15, 22)			0.868	
ALT (IU/L)	14 (9, 23)		15 (10, 21)			0.972	
T-Bil (mg/dL)	0.52 (0.45, 0.60)		0.52 (0.40, 0.64)			0.763	
WBC (10^3^/µL)	4.31 (3.86, 5.05)		5.34 (4.50, 6.32)			< 0.001	
Neutrophil (10^3^/µL)	2.70 (2.11, 3.33)		3.31 (2.62, 4.16)			< 0.001	
Lymphocyte (10^3^/µL)	1.24 (1.04, 1.52)		1.44 (1.14, 1.77)			0.002	
Monocyte (10^3^/µL)	0.25 (0.20, 0.29)		0.29 (0.24, 0.35)			< 0.001	
Hemoglobin (g/dL)	11.9 (11.3, 12.7)		12.4 (11.6, 13.2)			0.035	
Platelet (10^4^/µL)	26.7 (21.7, 29.6)		27.8 (22.8, 33.7)			0.063	
NLR	2.16 (1.58, 2.85)		2.26 (1.64, 3.20)			0.585	
PLR	19.4 (16.2, 28.9)		18.9 (15.0, 25.0)			0.188	
LMR	5.48 (3.67, 6.96)		5.00 (3.81, 6.25)			0.629	
Administration of G-CSF after day 15	30 (66.7)		36 (18.3)			< 0.001	
**Laboratory value on day 8 of TC therapy**							
WBC (10^3^/µL)	2.60 (2.25, 3.35)		3.63 (3.10, 4.47)			< 0.001	
Neutrophil (10^3^/µL)	1.48 (1.14, 2.07)		2.15 (1.68, 2.82)			< 0.001	
Lymphocyte (10^3^/µL)	0.95 (0.75, 1.13)		1.15 (0.94, 1.50)			< 0.001	
Monocyte (10^3^/µL)	0.04 (0.03, 0.06)		0.07 (0.05, 0.11)			< 0.001	
Hemoglobin (g/dL)	12.1 (11.2, 13.1)		12.5 (11.7, 13.4)			0.135	
Platelet (10^4^/µL)	20.0 (15.9, 23.3)		22.4 (18.1, 27.3)			0.007	
NLR	1.54 (1.15, 2.19)		1.89 (1.32, 2.61)			0.129	
PLR	19.4 (14.2, 32.3)		18.1 (13.9, 26.3)			0.323	
LMR	21.3 (14.4, 30.1)		15.0 (11.0, 22.5)			0.004	
Change of WBC (10^3^/µL)	–1.72 (–2.10, − 0.82)		–1.68 (–2.53, − 0.95)			0.650	
Change of neutrophil (10^3^/µL)	–1.10 (–1.57, − 045)		–1.12 (–1.83, − 0.40)			0.499	
Change of lymphocyte (10^3^/µL)	–0.21 (–0.47, − 0.09)		–0.28 (–0.48, − 0.08)			0.529	
Change of monocyte (10^3^/µL)	–0.21 (–0.23, − 0.14)		–0.21 (–0.27, − 0.15)			0.251	
Change of hemoglobin (g/dL)	0.2 (–0.4, 0.6)		0.1 (–0.5, 0.6)			0.348	
Change of platelet (10^4^/µL)	–6.0 (–9.0, − 3.1)		–5.9 (–8.6, − 2.6)			0.772	

Data are presented as medians (interquartile range) or frequencies (percentage)

Ccr, creatinine clearance; AST, aspartate aminotransferase; ALT, alanine aminotransferase; T-Bil, total bilirubin; WBC, white blood cell; NLR, neutrophil-lymphocyte ratio; PLR, platelet-lymphocyte ratio; LMR, lymphocyte-monocyte ratio

To elucidate the factors contributing to treatment suspension, we conducted a logistic regression analysis and formulated three models (Table 3). Model 1 incorporated solely pre-treatment laboratory values as covariates, Model 2 utilized laboratory values exclusively from day 8 of treatment, and Model 3 integrated both pre-treatment and day 8 laboratory values for a more comprehensive analysis. In Model 1, which investigated the relationship between pre-treatment laboratory values and treatment suspension, pre-treatment neutrophil and lymphocyte counts were identified as significant factors. In Model 2, which focused on laboratory values measured on day 8 of treatment, lymphocyte and monocyte counts emerged as significant factors. Significantly, in the comprehensive Model 3, which controlled for both time points, day 8 lymphocyte and monocyte counts remained highly significant independent predictors of treatment suspension, alongside pre-treatment neutrophil counts. This suggests that the early post-treatment changes captured on day 8 provide predictive information independent of the baseline hematological status.

**Table 3:** Logistic regression analysis assessing the association between treatment suspension and pre-treatment test value or laboratory value on day 8

	Univariate	Multivariate model 1	Multivariate model 2	Multivariate model 3
	OR (95% CI)	OR (95% CI)	OR (95% CI)	OR (95% CI)
**Patient characteristics**				
Age (per 10 years increase)	1.27 (0.90, 1.80)	1.41 (0.96, 2.07)		
**Pre-treatment laboratory values**				
Neutrophil on day 0 (per 1SD decrease)	2.39 (1.47, 3.89)	1.87 (1.07, 3.29)		1.90 (1.06, 3.42)
Lymphocyte on day 0 (per 1SD decrease)	2.01 (1.31, 3.07)	1.92 (1.19, 3.10)		
Monocyte on day 0 (per 1SDdecrease)	2.61 (1.61, 4.24)	1. 65(0.93, 2.93)		1.60 (0.86, 3.01)
Hemoglobin on day 0 (per 1SD decrease)	1.34 (0.97, 1.85)			
Platelet on day 0 (per 1SD decrease)	1.64 (1.07, 2.51)			
**Laboratory value on day 8 of TC therapy**				
Neutrophil on day 8 (per 1SD decrease)	2.02 (1.30, 3.13)		1.44 (0.94, 2.20)	
Lymphocyte on day 8 (per 1SD decrease)	2.05 (1.33, 3.14)		1.81 (1.13, 2.90)	1.87 (1.14, 3.06)
Monocyte on day 8 (per 1SDdecrease)	5.60 (2.64, 11.89)		3.95 (1.85, 8.41)	(2.01, 9.61)
Platelet on day 8 (per 1SD decrease)	1.79 (1.19, 2.70)		1.45 (0.93, 2.27)	
LMR on day 8 (per 1SD increase)	1.56 (1.17, 2.09)			

CI, confidence interval; LMR, lymphocyte-monocyte ratio; OR, odds ratio; SD, standard deviation

### Performance of a treatment suspension prediction model

**Fig. 2:**
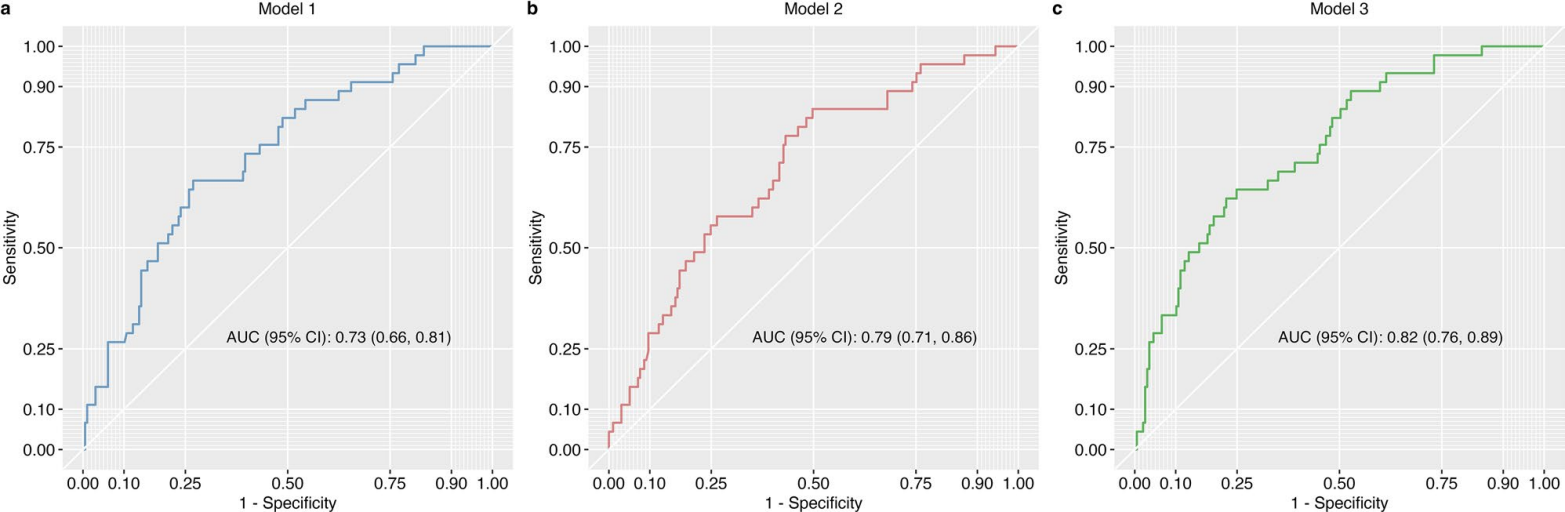
Comparison of predictive performance for treatment discontinuation detection using ROC analysis. (**a**) AUC of pre-treatment laboratory values (Model 1). (**b**) AUC of laboratory values on day 8 (Model 2). (**c**) AUC of laboratory values from pre-treatment and day 8 (Model 3)

ROC analysis was used to evaluate the effectiveness of predicting treatment suspension across the three models (Fig. 2). The predictive accuracies of the models were compared using AUC. Model 1, which employed pre-treatment values, achieved an AUC of 0.73 (95% confidence interval [CI]: 0.66–0.81). Model 2, which utilized day 8 values, demonstrated an AUC of 0.79 (95% CI: 0.71–0.86). Model 3, which incorporated both pre-treatment and day 8 values, attained the highest predictive performance with an AUC of 0.82 (95% CI: 0.76–0.89). Although Model 2 exhibited a higher AUC than Model 1, this increase did not reach statistical significance (*P* = 0.302). Crucially, Model 3 showed a statistically significant enhancement in the AUC compared with the pre-treatment only Model 1 (*P* = 0.011), validating the added predictive utility of early post-treatment monitoring.

Of the 242 patients analyzed, 180 (74.4%) had laboratory measurements taken exactly on day 8, forming a subgroup for the sensitivity analysis. The other patients had measurements within ± 3 days of day 8. Restricting the sensitivity analysis to the 180-patient subgroup preserved model performance: AUCs for Model 1, Model 2, and Model 3 were 0.71 (95% CI: 0.62–0.80), 0.80 (95% CI: 0.72–0.88), and 0.85 (95% CI: 0.78–0.91), respectively. As with the main analysis, Model 3 significantly outperformed Model 1 (*P* = 0.003), whereas Model 1 and Model 2 did not differ significantly (*P* = 0.144).

**Fig. 3:**
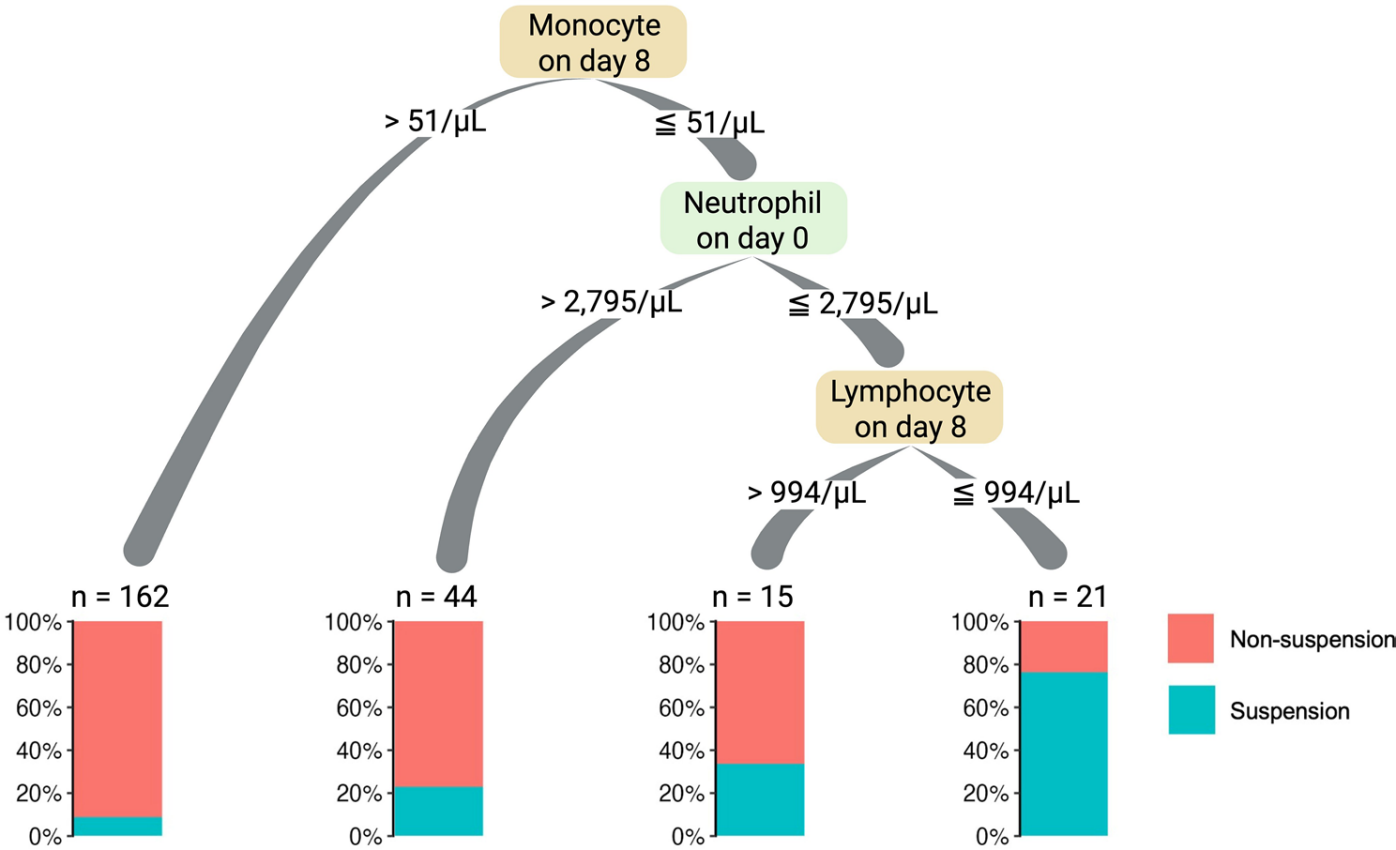
Decision tree analysis for the early detection of patients whose treatment is suspended

To derive clinically actionable thresholds from the most predictive Model 3, a decision tree analysis was conducted. This analysis identified key cutoff values for risk stratification: monocyte count on day 8 of treatment, 51/µL; lymphocyte count on day 8 of treatment, 994/µL; and pre-treatment neutrophil count, 2,795/µL (Fig. 3). Patients whose laboratory values were all below their corresponding cutoff values exhibited an increased risk of treatment discontinuation. Furthermore, a sensitivity analysis was performed by excluding the pre-treatment neutrophil count (day 0) to evaluate its contribution to the model. In this analysis, the decision tree failed to generate any significant splits, indicating that the day 0 neutrophil count is an essential factor for the construction of the predictive model.

## Discussion

The present study investigated the factors leading to discontinuation of the second cycle of TC therapy due to myelosuppression. The most significant finding is that incorporating post-treatment laboratory values obtained on day 8, in conjunction with baseline pre-treatment values, significantly improved the predictive accuracy. Specifically, the comprehensive model (Model 3) exhibited a statistically significant superiority (*P* = 0.011) in predictive performance compared to the model using pre-treatment factors alone (Model 1). The finding that pre-treatment neutrophil counts, in conjunction with monocyte and lymphocyte counts measured on day 8 post-treatment, are the strongest predictors, facilitates timely and reliable predictions of the likelihood of treatment suspension. This demonstrates that monitoring hematological changes at an early, actionable time point—specifically day 8—provides independent crucial information that complements baseline status. This finding supports the use of the combined model for more accurate individualized supportive care.

Model 1, a multivariate logistic model employing pre-treatment laboratory values, identified decreased neutrophil and lymphocyte counts prior to the initiation of treatment as significant risk factors for therapy suspension due to myelosuppression. In alignment with existing literature, lymphocyte and white blood cell counts at the commencement of treatment were recognized as risk factors for the development of myelosuppression [7–9], thereby corroborating previous findings. Although ratios such as lymphocyte-to-monocyte, platelet-to-lymphocyte, and neutrophil-to-lymphocyte have been proposed as prognostic biomarkers for inflammation in several meta-analyses [10–20], no significant differences were observed in these ratios between patients with and without treatment discontinuation. These results suggest that the decrease in neutrophil and lymphocyte counts observed in the treatment suspension group before therapy was not due to an inflammatory response but rather indicated that bone marrow function was already compromised at the onset of treatment. In other words, because the underlying cause differs from the poor prognosis associated with inflammation, appropriate dose adjustments and supportive care based on pre-treatment and early treatment blood cell counts may prevent treatment suspension and potentially improve the prognosis in patients at risk of discontinuing treatment.

Next, multivariate logistic regression analysis was conducted using the laboratory values from day 8 of treatment. When logistic regression was performed using only the day 8 post-treatment variables (Model 2), both lymphocyte and monocyte counts emerged as significant predictors. Furthermore, a logistic regression model that combined pre-treatment values with day 8 post-treatment measurements (Model 3) identified day 8 monocyte and lymphocyte counts, in addition to pre-treatment neutrophil counts, as statistically significant factors. Among the assessed models, Model 3 demonstrated the highest predictive accuracy, followed by Model 2. Notably, Model 3 exhibited a significantly superior predictive capability compared to Model 1. These findings suggest that post-treatment monocyte and lymphocyte counts may serve as valuable risk indicators for treatment suspension compared to pre-treatment values. The incorporation of day 8 post-treatment values into the risk stratification process appears to be particularly advantageous for maintaining treatment intensity and informing early intervention strategies for patients with gynecologic malignancies. However, it is crucial to acknowledge that none of the patients in this study received G-CSF on day 8 of treatment. Consequently, the efficacy of administering G-CSF to patients exhibiting monocyte and lymphocyte counts below the respective reference thresholds identified in this investigation (monocyte count: 51/µL; lymphocyte count: 994/µL) on day 8 in maintaining treatment intensity remains unclear. Nevertheless, the early identification of individuals susceptible to myelosuppression by day 8 following treatment initiation offers valuable information for optimizing subsequent monitoring. This approach may ultimately facilitate the prevention of severe adverse events such as FN.

Our decision tree analysis identified specific cutoff values for monocytes and lymphocytes on day 8 and for pre-treatment neutrophils, which can be interpreted based on their biological characteristics and institutional reference ranges. First, monocytes have a short circulating lifespan (1–3 days), and their precursors exhibit rapid turnover, making them highly sensitive indicators of chemotherapy-induced marrow stress [21]. Our hospital’s reference range for monocytes is 100–480/µL. The identified cutoff of 51/µL is approximately half the lower limit of normal (LLN), indicating profound depletion. Since monocyte recovery typically precedes neutrophil recovery [22], a failure to rise above this threshold by day 8 likely signals a protracted delay in hematopoietic regeneration. Thus, the day 8 monocyte count serves as a specific, early warning sign of the bone marrow’s inability to cope with TC therapy toxicity. Second, regarding lymphocytes (reference range: 900–2,980/µL), the cutoff of 994/µL falls within the normal range but hovers near the lower limit. While lymphocytes generally have longer lifespans, a decline to this borderline level as early as day 8 indicates significant immediate hematologic toxicity and contributes to the overall risk assessment. Third, the pre-treatment neutrophil cutoff was identified at 2,795/µL (reference range: 1,170–5,780/µL). Although this value is well above the LLN, it functions as an indicator of ‘bone marrow reserve capacity.’ A higher baseline count implies a robust marrow reserve capable of withstanding cytotoxic stress. This finding aligns with previous reports identifying baseline neutrophil thresholds (e.g., 2,000–3,500/µL) as predictors for febrile neutropenia or severe toxicity in ovarian and other cancers [23–25]. The clinical importance of baseline neutrophil counts was further supported by a sensitivity analysis; when day 0 neutrophil counts were excluded from the decision tree analysis, the algorithm failed to generate any significant splits. This indicates that the day 8 dynamic response must be interpreted in the context of the initial bone marrow reserve to accurately predict treatment outcomes, and that baseline status is an essential prerequisite for the construction of our predictive model. Consistent with Shimanuki et al., who reported that combining pre-treatment neutrophil and monocyte counts improves risk prediction [25], our study confirms that integrating day 8 dynamics with baseline reserve capacity offers the most robust prediction for treatment delays.

The ability to identify high-risk individuals by day 8, using a threshold such as a monocyte count below 51/µL, provides a critical window for intervention. Although none of the patients in this study received G-CSF on day 8, therapeutic G-CSF administration by day 15 may prove ineffective in preventing treatment delays. In this study, the overall incidence of FN was low (only 3 patients, < 1%). This is notably lower than the 8% incidence reported by du Bois et al. [26] in ovarian cancer patients receiving TC therapy. The low incidence in our cohort is likely attributable to the therapeutic administration of G-CSF on day 15, which successfully prevented severe infection but failed to prevent the treatment delay. Therefore, the primary value of early identification at day 8 lies in optimizing monitoring to maintain treatment intensity. This approach directly supports clinical pharmacy practice by enabling pharmacists to proactively identify patients at high risk of dose delay/discontinuation based on day 8 test results. Pharmacists can use these specific cutoff values to counsel patients, alert physicians, and recommend timely supportive interventions, such as dose adjustment or the prophylactic administration of G-CSF before the intended day 21 cycle. By adopting this monitoring strategy, healthcare teams can move from reactive management to proactive risk mitigation, contributing to improved RDI.

This study focuses on day 8 for two primary reasons. First, as stated in the result section, the treatment suspension group had a significantly higher proportion of patients receiving G-CSF after day 15. This suggests that intervention to prevent treatment suspension would be too late at that point. Second, in our hospital’s department of obstetrics and gynecology, blood tests are performed weekly after treatment. Consequently, the laboratory results uniformly available for all patients are those from day 0 or day 1, and then day 8 and day 15. While laboratory values at other time points might potentially be more beneficial predictors, considering practical application in clinical practice, day 8 was deemed the most appropriate.

Our findings regarding monocyte counts warrant comparison with previous studies. For instance, Kondo et al. reported a marked difference in monocyte counts on days 6–8 between patients who developed grade 3/4 neutropenia (87/µL) and those with grade 1/2 neutropenia (248/µL) in lung cancer patients [27]. Furthermore, they noted that all patients with monocyte counts < 150/µL developed severe neutropenia. In contrast, the difference observed in our study (40/µL in the suspension group vs. 70/µL in the non-suspension group) appears numerically smaller. This discrepancy is primarily attributable to the difference in study endpoints. While previous studies focused on the onset of neutropenia, our study focused on treatment suspension. In our cohort, 84 patients (42.6%) in the non-suspension group still experienced grade 3/4 neutropenia. Because a significant portion of the control group experienced severe myelosuppression, their monocyte counts were also depressed, leading to a smaller inter-group difference compared to studies comparing neutropenic vs. non-neutropenic patients. However, this does not diminish the clinical value of the findings. The novelty of this study lies in demonstrating that even within a population experiencing widespread myelosuppression, a specific threshold (day 8 monocyte count ≤ 51/µL) can effectively discriminate patients who will fail to recover in time for the next cycle. This identifies day 8 as a critical window for interventions focused on maintaining RDI in gynecologic malignancies.

This study had several limitations. First, its retrospective, single-center design limits the generalizability of our findings. Detailed information on established risk factors for myelosuppression—such as performance status, specific comorbidities, and concomitant medications—could not be obtained uniformly for all patients. While we evaluated nutritional status using BMI and albumin levels (which were not identified as significant predictors), the inability to adjust for performance status and other potential clinical confounders remains a limitation. The interpretation of our results must account for the potential influence of these unmeasured factors. Second, the timing of blood tests was dictated by the institutional weekly schedule (days 0/1, 8, and 15), with no routine measurements on intermediate days (e.g., days 5 or 10) that might theoretically provide additional predictive value. Although this study focused on laboratory values obtained on day 8, not all patients had blood tests performed exactly on that day because of the retrospective nature of the outpatient setting. To address this temporal variability, we conducted a sensitivity analysis restricted to patients whose laboratory measurements were obtained exactly on day 8. This analysis showed that the predictive performance was preserved and even slightly improved compared with the main analysis. This indicates that timing variability had a limited impact on the study’s conclusions. Day 8 was selected as the earliest and most practical time point for intervention, a choice supported by the model’s robust predictive performance. However, it represents a limitation that no participants received prophylactic G-CSF on day 8 in this cohort. Therefore, although low monocyte (≤ 51/µL) and lymphocyte (≤ 994/µL) counts showed strong predictive signals, the clinical efficacy of G-CSF intervention at this specific time point remains to be validated. Third, 66 patients received therapeutic G-CSF on or after day 15. This intervention was typically administered as a continuous 3-day course starting on day 15, or continued until the neutrophil count recovered to ≥ 1,000/µL based on physician discretion. While the resultant neutrophil recovery likely influenced decisions regarding cycle suspension, the retrospective design precluded a definitive quantification of this influence. Future research should focus on prospective multicenter studies to validate the utility of day 8 monocyte counts as a guide for individualized supportive care strategies.

## Conclusions

This study identified early post-treatment hematological indices, particularly the day 8 monocyte and lymphocyte counts, as highly significant risk factors for patients unable to proceed with subsequent cycles of TC therapy for gynecological cancer due to myelosuppression. Crucially, the predictive model incorporating these day 8 factors demonstrated statistically superior performance compared to models based on pre-treatment values alone. These findings highlight the critical importance of systematic hematologic monitoring, specifically focusing on the day 8 monocyte and lymphocyte count as a superior and actionable biomarker, to ensure the maintenance of treatment intensity and strict adherence to established drug administration schedules. To optimize therapeutic efficacy and enhance patient quality of life, healthcare professionals should utilize these day 8 markers for proactive risk stratification and timely implementation of supportive care measures.

## Data Availability

The datasets used and analysed during the current study are available from the corresponding author on reasonable request.

## Abbreviations

AUC: Area Under the Curve
CI: Confidence Interval
CTCAE: Common Terminology Criteria for Adverse Events
FN: Febrile Neutropenia
Peg-G-CSF: Polyethylene glycol-conjugated Granulocyte Colony-Stimulating Factor
RDI: Relative Dose Intensity
ROC: Receiver Operating Characteristic
TC: Paclitaxel plus Carboplatin
